# P-7. Characterization of Blood Culture Ordering Practices Pre and Post Blood Culture Menu Implementation at a Single Veterans Affair Medical Center

**DOI:** 10.1093/ofid/ofaf695.238

**Published:** 2026-01-11

**Authors:** Tina H Dao, Cassidy Stegall, Nicholas Liang, Jarred Bowden, Anna Mitchell, Peter Zhang, Louis Yn, Jessica G Bennett

**Affiliations:** University of Tennessee Health Science Center College of Medicine, Memphis, TN, Memphis, Tennessee; University of Tennessee Health Science Center, Memphis, Tennessee; University of Tennessee Health Science Center, Memphis, Tennessee; Memphis VA Medical Center, Memphis, Tennessee; Lt. Col. Luke Weathers, Jr. Veterans Affairs Medical Center, Memphis, TN, Memphis, Tennessee; Children’s Hospital of Philadephia, University of Pennsylvania, Philadephia, Pennsylvania; University of Tennessee Health Science Center College of Medicine, Memphis, TN, Memphis, Tennessee; VAMC Memphis, Germantown, Tennessee

## Abstract

**Background:**

Blood culture (BCx) is the gold standard for definitive diagnosis of bacteremia and fungemia, but there is limited guidance for ordering practices. Unnecessary BCxs increase the risk of false positives, leading to increase length of stay, inappropriate antibiotic use, and additional testing. Diagnostic stewardship aims to optimize testing and improve patient management and outcomes. This study aimed to describe BCx ordering practices at a single facility before and after implementation of a BCx ordering menu.

**Figure 1.**
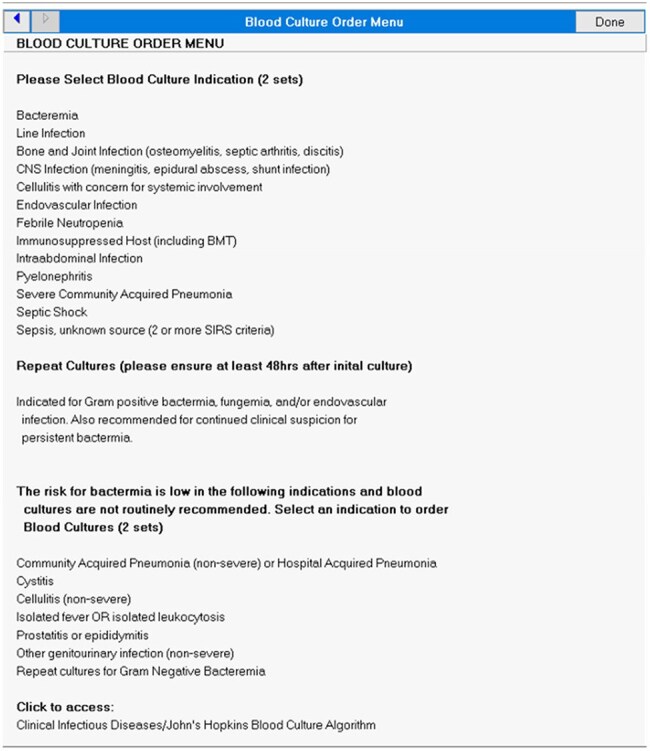
Blood Culture Ordering Menu

**Figure 2.**
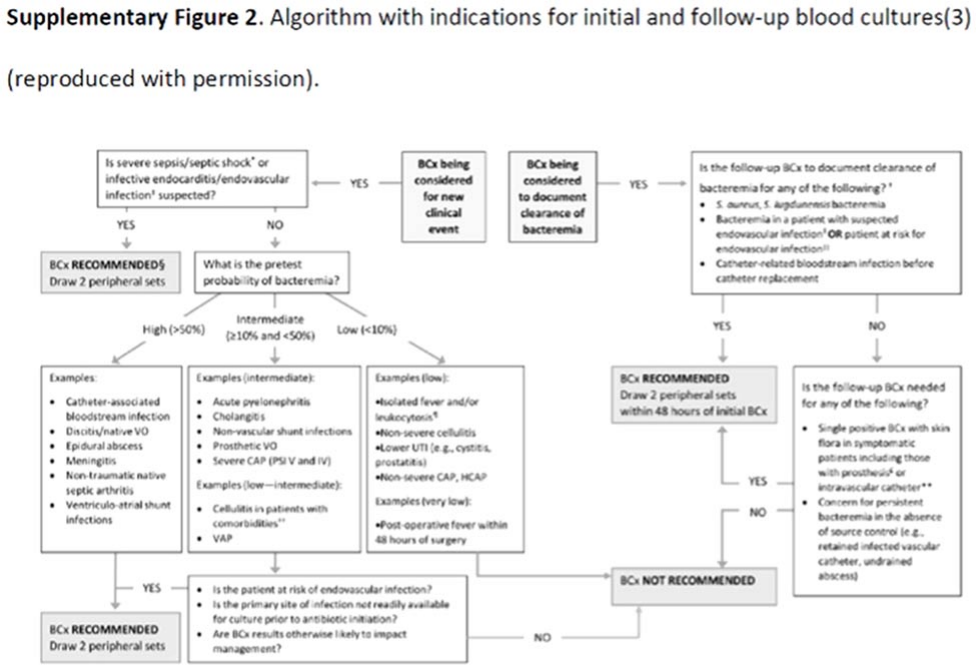
Algorithm with indications for initial and follow-up blood cultures (reproduced with permission) Valeria Fabre, Sima L Sharara, Alejandra B Salinas, Karen C Carroll, Sanjay Desai, Sara E Cosgrove, Does This Patient Need Blood Cultures? A Scoping Review of Indications for Blood Cultures in Adult Nonneutropenic Inpatients, Clinical Infectious Diseases, Volume 71, Issue 5, 1 September 2020, Pages 1339–1347, https://doi.org/10.1093/cid/ciaa039

**Methods:**

Our facility implemented a BCx ordering menu on 09/09/24, in which providers must select an ordering indication, shown in Figure 1. This was a pre-and-post retrospective review of BCx ordered PRE (11/01/23 to 12/31/23), and POST (11/01/24 to 12/30/24) implementation. BCxs were excluded if there was no encounter 5 days before or 2 days after collection or initial BCxs were drawn at another facility. Labs, vitals, order date/time, provider, location, and results were extracted from the Corporate Data Warehouse. Indications for BCx were determined via chart review. BCx appropriateness was determined by an algorithm denoting low, intermediate, or high bacteremia risk shown in Figure 2. Intermediate and high risk were deemed appropriate. Endpoints were analyzed with descriptive statistics.

**Figure 3:**
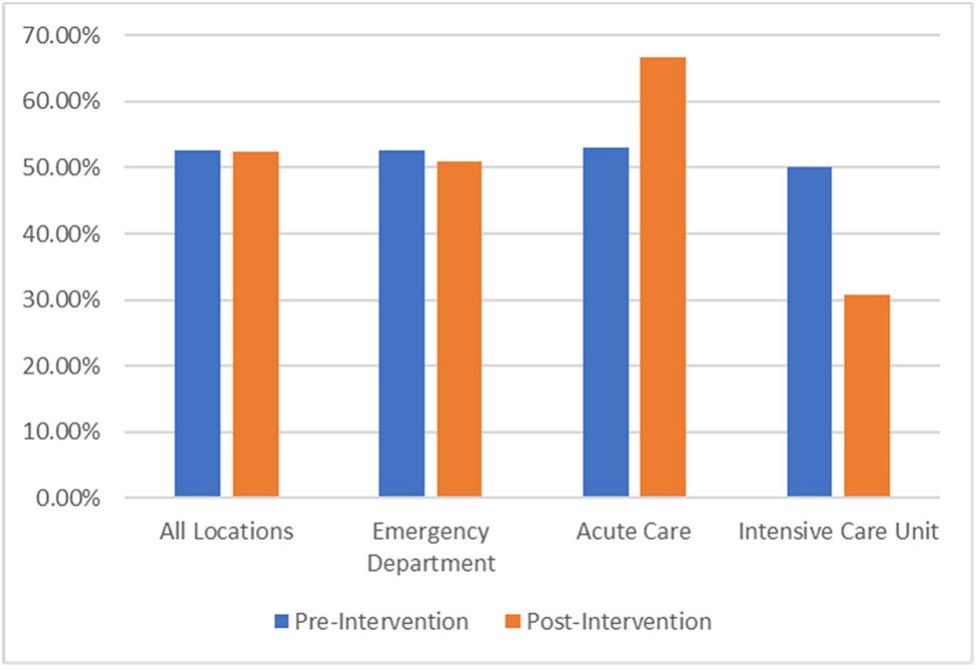
Appropriateness of Blood Cultures PRE and POST- intervention

**Table 1.**
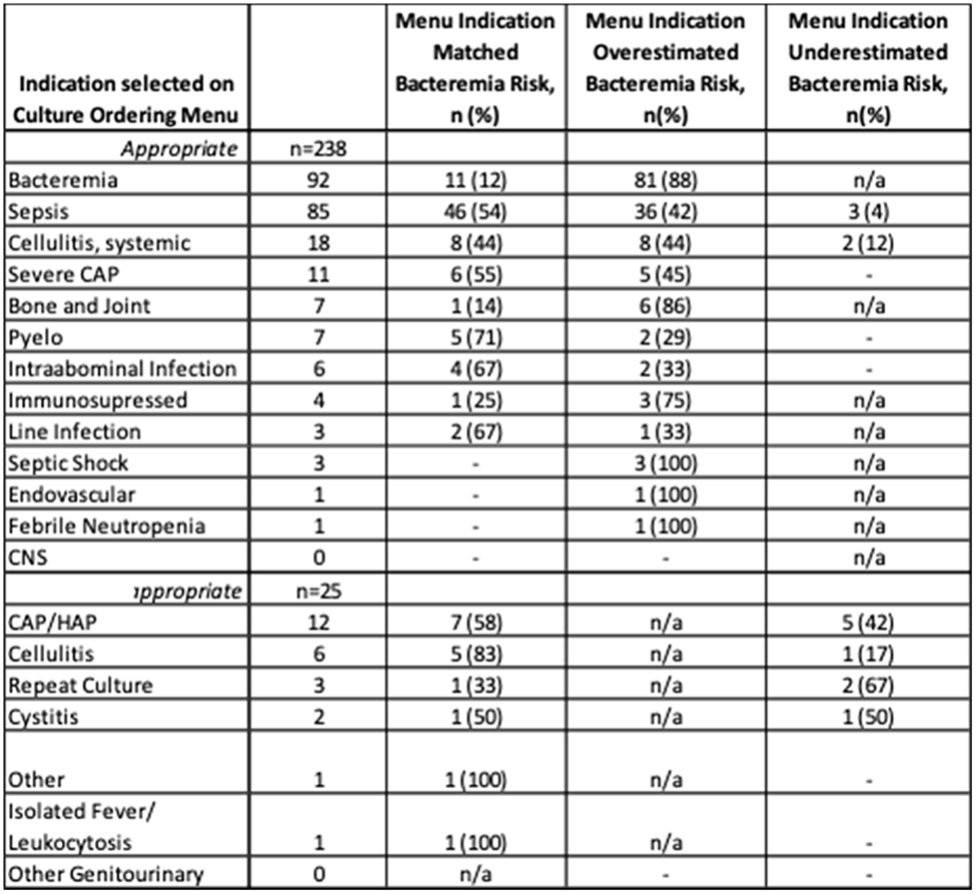
Culture Ordering Menu Indication Selections compared to Indications documented in Electronic Medical Record

**Results:**

243/338 BCx ordered were evaluated and 215 were analyzed in the PRE-group and 298 BCx were evaluated and 273 analyzed in the POST group. Overall, appropriateness was not different between the two groups (53% vs 52%, p=0.96) (Figure 3). Interestingly, when evaluated per indication chosen from the culture ordering menu, POST appropriate rates were >90% for all locations. The most common indications selected on the menu were bacteremia (n=92) and sepsis (n=85) (Table 1). There were low positivity rates for BCx in both groups (7.9% PRE, 8% POST).

**Conclusion:**

This study found no difference in rate of appropriate BCx ordering after the implementation of a BCx ordering menu. Our evaluation also found that the order menu overestimated appropriateness, likely due to confusion regarding the indication “bacteremia”. Our findings have prompted plans for a third phase of this project, including order menu updates, re-education, and audit and feedback.

**Disclosures:**

All Authors: No reported disclosures

